# The relationship between previous hamstring injury and the concentric isokinetic knee muscle strength of irish gaelic footballers

**DOI:** 10.1186/1471-2474-9-30

**Published:** 2008-03-06

**Authors:** Kieran O'Sullivan, Brian O'Ceallaigh, Kevin O'Connell, Amir Shafat

**Affiliations:** 1 Physiotherapy Department, University of Limerick, Limerick, Ireland; 2 Department of Physical Education and Sports Sciences, University of Limerick, Limerick, Ireland; 3 Physical Activity, Occupation and Health Research Unit, University of Limerick, Limerick, Ireland

## Abstract

**Background:**

Hamstring injury is one of the most common injuries affecting gaelic footballers, similar to other field sports. Research in other sports on whether residual hamstring weakness is present after hamstring injury is inconsistent, and no study has examined this factor in irish gaelic footballers. The aim of this study was to examine whether significant knee muscle weakness is present in male Irish gaelic footballers who have returned to full activity after hamstring injury.

**Methods:**

The concentric isokinetic knee flexion and extension strength of 44 members of a university gaelic football team was assessed at 60, 180 and 300 degrees per second using a Contrex dynamometer.

**Results:**

Fifteen players (34%) reported a history of hamstring strain, with 68% of injuries affecting the dominant (kicking) limb. The hamstrings were significantly stronger (p < 0.05) on the dominant limb in all uninjured subjects. The previously injured limbs had a significantly lower (p < 0.05) hamstrings to quadriceps (HQ) strength ratio than all other non-injured limbs, but neither their hamstrings nor quadriceps were significantly weaker (p > 0.05) using this comparison. The previously unilaterally injured hamstrings were significantly weaker (p < 0.05) than uninjured limbs however, when matched for dominance. The hamstring to opposite hamstring (H:oppH) strength ratio of the previously injured players was also found to be significantly lower (p < 0.05) than that of the uninjured players.

**Conclusion:**

Hamstring muscle weakness was observed in male Irish gaelic footballers with a history of hamstring injury. This weakness is most evident when comparisons are made to multiple control populations, both within and between subjects. The increased strength of the dominant limb should be considered as a potential confounding variable in future trials. The study design does not allow interpretation of whether these changes in strength were present before or after injury.

## Background

Gaelic football is one of the major national field sports in Ireland. Similar to other field sports played at high speed and intensity, a significant rate of injury has been observed in gaelic football [1,2]. Hamstring injury has been shown to account for approximately 13% of all injuries among gaelic footballers [3,4]. A similarly high rate of hamstring injury has also been found among athletes in other sports involving kicking and running, such as Australian rules football [5]. This rate of injury is highlighted by the amount of match playing time missed by players, with hamstring injury alone resulting in approximately 21 missed player games per club per year in Australian football [5].

There have been several factors hypothesised to contribute to the risk of hamstring injury. These include inadequate warm-up, fatigue, previous injury, knee muscle weakness or strength imbalance, increasing age, poor movement discrimination, poor flexibility, increased lumbar lordosis and poor running technique [6-8]. While there is emerging evidence that the cause of hamstring injury may be multifactorial [8,9], one potential contributing factor which has been much researched is muscle weakness [10-12]. As well as looking at risk factors prospectively [11], there have been many retrospective trials trying to identify potential deficits present in athletes after hamstring injury [13-15]. The results of these trials have been contradictory and inconsistent. Many retrospective studies, across a variety of sports, have found that athletes with a history of hamstring injury had significantly reduced thigh muscle strength and significant strength imbalance when compared to athletes with no history of hamstring injury [13,15,16]. However, other retrospective studies have found no such relationship between previous hamstring injury and muscle strength [14,17]. In addition, in two prospective studies carried out among Australian football players [11,12], it was found that those with pre-season muscle weakness and strength imbalance were at a significantly greater risk of sustaining a hamstring strain. These results were, however, directly contradicted by another prospective trial [10], which found no such association. The reasons for these inconsistencies, in both retrospective and prospective research, are largely unclear. Methodological differences and differences in study populations may explain part of this. It is possible that further sources of confusion are that studies do not all make the same comparisons (within-subject or between-subject), and do not all account for the potential effect of limb dominance affecting the results [10-13].

In addition, strength testing protocols have varied between concentric, isometric and eccentric [11,15,18]. It has been suggested that eccentric hamstring strength may be more sensitive at detecting changes after injury [15,19], however the increased risk of injury [20] is a concern when dealing with previously injured subjects. Therefore, the current study used a more demanding concentric testing protocol than those used in previous studies, to try to avoid the need for eccentric testing.

There has been no previously published research on isokinetic strength after hamstring injury in gaelic footballers, despite the high prevalence of the injury. Most previous studies on gaelic footballers have been primarily concerned with the incidence of injuries [2,21,22]. The aim of this retrospective study was to determine whether significant knee muscle weakness was present among Irish male gaelic football players with a history of hamstring injury. This could help clarify which rehabilitation strategies might be justified in the management of such players, especially since previous hamstring injury is the biggest risk factor for future hamstring injury [8]. Additional aims were to clarify how any potential changes in muscle strength after injury were best identified and to take into account the potential effect on muscle strength of limb dominance.

## Methods

### Participants

50 subjects were recruited from the University of Limerick senior male gaelic football panel, and screened for entry into the study. Players were excluded if they were less than 18 years of age, had sustained a hamstring injury in the 12 weeks immediately prior to testing, or if they reported any current lower extremity injury that may have limited their ability to perform maximal voluntary contractions. After screening, 44 subjects were eligible for the study. All subjects gave written informed consent. Their mean (± SD) age was 21.2 (± 1.8) years, weight was 82.1 (± 8.6)kg and height was 180.5 (± 6.6)cm.

### Procedure

The Clinical Therapies Research Ethics Committee of the University of Limerick approved the study. The testing procedure took place in the clinical therapies research laboratory of the University of Limerick. Prior to testing subjects were questioned regarding their hamstring injury history for the previous 12 months. History of hamstring injury was noted from the subject's own subjective report. A hamstring muscle strain was considered significant to the study if it prevented the subject from participating in a match or caused them to miss training for a period of one week or more. A definition similar to this has been used in previous studies [13,14]. Limb dominance was defined as preferred kicking leg [12]. Subjects were requested not to train or exercise vigorously during the four hours preceding testing. All testing consisted of a warm up, the actual test protocol, and a cool down. Warm up and cool down consisted of 10 minutes of stationary cycling using low resistance and a moderate speed, followed by stretching of the hamstrings and quadriceps muscles. Maximum voluntary concentric torque of the hamstrings and quadriceps was assessed using a ConTrex-MJ isokinetic dynamometer (ConTrex AG, Dubendorf, Switzerland). This dynamometer has been shown to be reliable for assessment of isokinetic knee flexion and extension strength [23-25]. Subjects were tested in the seated position as recommended by the dynamometer manufacturers and were secured by a seatbelt system consisting of stabilisation straps. The axis of rotation of the dynamometer was aligned with the centre of the lateral femoral condyle and the resistance pad at the end of the lever arm was positioned two centimetres proximal to the lateral malleolus. Each subject's reciprocal concentric knee flexion (hamstrings) and extension (quadriceps) torque was measured at angular velocities of 60, 180 and 300 degrees per second (°/sec), similar to previous research [11]. Torque was measured through a predetermined range of knee motion within safety limits specific to each subject. Subjects initially performed three submaximal trial repetitions at each of the three angular velocities in order to familiarise themselves with the testing procedure. The actual test procedure consisted of three sets of six maximal contractions at each velocity. A 90 second recovery period was allowed between sets, and a 180 second rest period between angular velocities. Therefore, each participant completed a total of 108 maximum contractions during the trial. The order of leg testing was randomised, however the quadriceps was tested before the hamstrings. All subjects were tested bilaterally. All torques were corrected for the effects of gravity [10]. Subjects were given advance verbal instructions and encouragement to push as hard as possible, to facilitate maximal effort during testing. Subjects were not given any additional verbal or visual feedback during the test.

### Statistical Analysis

The data was initially tested to ensure it was normally distributed. Paired t-tests were used to analyse the strength of dominant and non-dominant limbs, and to examine within-subject differences in unilaterally injured subjects. Independent t-tests were used to compare the previously injured and non-injured limbs between subjects. The isokinetic variables assessed were average peak torque (APT) and average peak torque normalised for bodyweight (APTBW). In addition, muscle strength ratios (i.e. hamstrings-to-quadriceps (H:Q) and hamstrings-to-opposite hamstrings (H:oppH) ratios) were analysed. A significance level of p < 0.05 was set for all tests. All data was analysed using SPSS 15.0 for Microsoft Windows.

## Results

15 players (34%) reported a history of hamstring injury, 4 of these players being injured bilaterally, resulting in a total of 19 injured limbs. Each injured subject sustained one injury each in that time. 13 of the injuries (68%) occurred on the subject's dominant (kicking) limb. Each reported hamstring injury resulted in a mean (± SD) of 3.5 (± 1.8) weeks missed playing time.

### Comparison of Dominant to Non-Dominant Limbs

**Figure 1:**
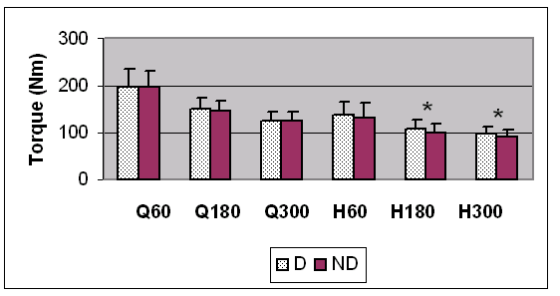
Comparison of quadriceps (Q) and hamstrings (H) average peak torque values for dominant (D) and non-dominant (ND) uninjured subjects (n = 29) at 60°/sec, 180°/sec and 300°/sec. * indicates a statistically significant difference (p < 0.05).

For the uninjured subjects (n = 29) the dominant limb hamstrings were significantly stronger at 180°/sec and 300°/sec (APT, APTBW) (see Figure 1). In addition, the HQ ratio in the dominant limb was also significantly higher at these 2 speeds. For the entire study group (n = 44) however, the dominant limb hamstrings were only significantly stronger at 180°/sec (APT, APTBW). There was no significant difference in quadriceps strength between dominant and non-dominant limbs.

### Comparison of all injured limbs (n = 19) and all uninjured limbs (n = 69)

The HQ ratio was significantly reduced at 60°/sec (p < 0.05). Hamstring strength was reduced in the previously injured limbs at 60°/sec, but this did not reach statistical significance (see Table 1). No other significant differences were found.

**Table 1:** Comparison of all previously injured and all uninjured limbs. Mean (SD).

Variable	Injured Limbs n = 19	Uninjured Limbs n = 69
Hapt_60 _(Nm)	119.2 (28.7)	132.2 (29.1)
Hapt_180 _(Nm)	101.4 (20.0)	103.3 (20.2)
Hapt_300 _(Nm)	93.2 (18.4)	92.4 (16.7)
Qapt_60 _(Nm)	191.8 (43.6)	193.1 (38.0)
Qapt_180 _(Nm)	149.4 (32.2)	147.2 (26.0)
Qapt_300 _(Nm)	130.4 (29.2)	124.4 (21.2)
Hptbw_60 _(Nm/kg)	1.5 (0.3)	1.6 (0.3)
Hptbw_180 _(Nm/kg)	1.2 (0.2)	1.3 (0.2)
Hptbw_300 _(Nm/kg)	1.1 (0.2)	1.1 (0.2)
Qptbw_60 _(Nm/kg)	2.3 (0.5)	2.4 (0.5)
Qptbw_180 _(Nm/kg)	1.8 (0.4)	1.8 (0.3)
Qptbw_300 _(Nm/kg)	1.6 (0.3)	1.5 (0.3)
H:Q_60_	0.62 (0.1)	0.69 (0.1) *
H:Q_180_	0.69 (0.1)	0.71 (0.1)
H:Q_300_	0.73 (0.1)	0.75 (0.1)

H = Hamstrings, Q = Quadriceps, apt = Average peak torque, ptbw = Average peak torque per kilogram bodyweight, Nm = Newton metre, Nm/kg = Newton metre per kilogram bodyweight, H:Q = hamstring-to-quadriceps ratio, * = statistically significant (p < 0.05).

### Comparison of injured (n = 13) and uninjured (n = 29) dominant limbs

**Figure 2:**
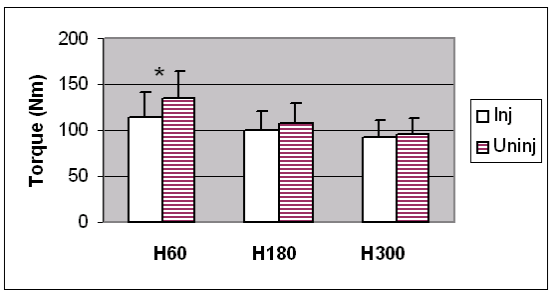
Comparison of hamstrings (H) average peak torque values between previously injured (Inj) (n = 13) and uninjured (Uninj) (n = 29) dominant limbs at 60°/sec, 180°/sec and 300°/sec. * indicates a statistically significant difference (p < 0.05).

The injured dominant hamstrings were significantly weaker than the uninjured 'matched' dominant hamstrings (APT, APTBW) at 60°/sec (see Figure 2). In addition, the HQ ratio was significantly lower at 60°/sec. No other significant differences were found (See Table 2).

**Table 2:** Comparison of previously injured dominant limbs and 'matched' uninjured dominant limbs. Mean (SD).

Variable	Previously Injured n = 13	'Matched' Uninjured n = 29
Hapt_60_(Nm)	115.3 (27.0)	136.2 (28.0) *
Hapt_180_(Nm)	99.3 (21.3)	107.9 (20.7)
Hapt_300_(Nm)	91.7 (20.3)	95.6 (17.1)
Qapt_60_(Nm)	190.2 (44.7)	196.9 (37.1)
Qapt_180_(Nm)	146.0 (36.9)	149.4 (25.5)
Qapt_300_(Nm)	127.4 (32.0)	124.9 (19.0)
Hptbw_60_(Nm/kg)	1.4 (0.3)	1.7 (0.3) *
Hptbw_180_(Nm/kg)	1.2 (0.2)	1.3 (0.2)
Hptbw_300_(Nm/kg)	1.1 (0.2)	1.2 (0.2)
Qptbw_60_(Nm/kg)	2.3 (0.5)	2.4 (0.4)
Qptbw_180_(Nm/kg)	1.8 (0.4)	1.9 (0.3)
Qptbw_300_(Nm/kg)	1.5 (0.3)	1.5 (0.2)
H:Q_60_	0.61 (0.1)	0.69 (0.1) *
H:Q_180_	0.69 (0.1)	0.73 (0.1)
H:Q_300_	0.74 (0.1)	0.77 (0.1)

H = Hamstrings, Q = Quadriceps, apt = Average peak torque, ptbw = Average peak torque per kilogram bodyweight, Nm = Newton metre, Nm/kg = Newton metre per kilogram bodyweight, H:Q = hamstring-to-quadriceps ratio, * = statistically significant (p < 0.05).

### Unilaterally injured players (n = 11)

**Figure 3:**
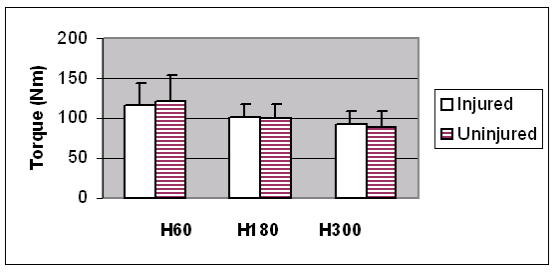
Within-subject comparison of hamstrings average peak torque values between unilaterally injured (n = 11) and uninjured (n = 29) limbs at 60°/sec, 180°/sec and 300°/sec. There was no statistically significant difference (p > 0.05).

**Figure 4:**
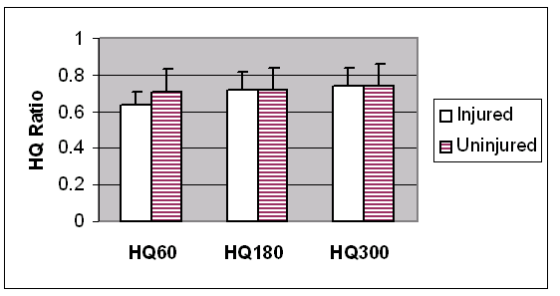
Within-subject comparison of hamstrings to quadriceps (HQ) strength ratios between unilaterally injured (n = 11) and uninjured (n = 29) limbs at 60°/sec, 180°/sec and 300°/sec. There was no statistically significant difference (p > 0.05).

Hamstrings strength and HQ ratios were reduced in the previously injured limbs at 60°/sec, however these differences did not reach statistical significance (see Figures 3 and 4). The H:oppH ratio was however significantly reduced in the unilaterally injured players at 60°/sec and 180°/sec, when compared to the uninjured subjects (see Table 3).

**Table 3:** Comparison of hamstrings-to-opposite hamstrings (H:oppH) ratios between the unilaterally injured (n = 11) and the uninjured subjects (n = 29). Mean (SD).

Variable	Previously Injured n = 11	Uninjured n = 29	p value
H:oppH_60_	0.91 (0.13)	1.05 (0.15)	0.011*
H:oppH_180_	0.99 (0.14)	1.09 (0.13)	0.024*
H:oppH_300_	0.98 (0.14)	1.06 (0.09)	0.051

* = statistically significant (p < 0.05)

## Discussion

No published research currently exists examining the relationship between hamstring injury and muscular strength in gaelic footballers. McIntyre & Hall [26] previously measured knee muscle concentric strength amongst a similar population of university gaelic footballers. They did not relate the torque measurements, expressed as PTBW, to any history of hamstring strain, however the strength values they obtained were broadly similar to those in the current study. The actual torque values from both studies of amateur gaelic football teams appear to be lower than that previously described for professional athletes in other sports, both for APT and APTBW [10-13,15]. Despite this, the muscle strength ratios observed in this study (HQ, H:oppH) are relatively consistent with published trials in other sports.

This study compared the previously injured limbs to a number of suitable controls, in an attempt to get an accurate picture of the relationship between previous injury and muscle strength. Many previous trials had only compared subjects between-subjects and not within-subject [10,13]. As previous research has demonstrated that the isokinetic strength of the injured players uninjured limb may be reduced [15], it was important to have a number of normative comparisons, rather than examining only between-subject or within-subject differences. For example, the current study has shown how some comparisons (Table 1) make it appear as though strength is not significantly reduced in limbs which have been previously injured, while other comparisons do (Table 2). The current study also considered the potential effect of limb dominance, when comparing with uninjured subjects, which has been considered in some [14] but not all [17] previous similar trials. Some previous trials have used the average of the uninjured subjects bilateral values as a comparison, however this was unsuitable in this trial due to the higher strength of the dominant limbs, and the higher proportion of dominant limbs in the injured group.

### Dominance

This study found that the dominant limb hamstrings were stronger than the non-dominant hamstrings. This could be due to the kicking involved in gaelic football, in agreement with recent studies showing a dominance effect in female gaelic footballers [27], as well as other studies [28] demonstrating significant asymmetry between dominant and non-dominant limbs. Other studies however have not found this dominance effect in numerous populations, even when similar definitions of leg dominance were used [10,29,30]. In the current study, 68% of the injuries sustained occurred on the subject's dominant limbs. Previous studies have reported dominant limb injury rates of between 37.5 – 71% [10,31]. The dominant limb injury rate found in this study is at the higher end of this scale. In contrast, Orchard et al. [11] & Cameron et al. [12] found no significant correlation between leg dominance and hamstring injury occurrence among Australian football players. The reason for these conflicting results is unclear. There may be a particular sports-specific link between hamstring injury and dominance in gaelic football, especially since 19% of hamstring injuries may occur during kicking [32]. The fact that the dominant limbs were stronger, and that they were more likely to be injured, meant dominance had to be taken into account when analysing data, by matching injured dominant limbs to non-injured dominant limbs.

### Relationship between strength and previous injury

This study demonstrated some evidence of hamstring muscle weakness and muscle strength imbalance in gaelic footballers after previous hamstring injury. This was identified in a number of ways;

• Reduced HQ ratio at 60°/sec when all injured limbs were compared to all healthy limbs

• Reduced hamstrings strength and reduced HQ ratio at 60°/sec for previously injured dominant limbs, compared to matched uninjured dominant limbs

• Reduced H:oppH ratio at 60°/sec and 180°/sec between the unilaterally injured players and the uninjured players

• The unilaterally injured hamstrings tended (non-significant) to be weaker, rather than stronger, when compared within-subject. This was an interesting finding, since 9 of the 11 (82%) injuries were on the dominant limb. Given this predominance of dominant limbs, the injured group would have been expected to have stronger hamstrings if injury was not related to hamstring strength.

Some previous retrospective studies, across a variety of sports, have also demonstrated hamstrings weakness or muscle strength imbalance after injury, similar to the current study [13,15,16]. However, other retrospective studies found no such relationship between previous hamstring injury and weakness or muscle strength imbalance [10,14,15,17,33]. Some of these trials which question the relationship between previous injury and weakness appear to display a non-significant differences between the groups [14,15,33]. It may be that the relatively demanding protocol used in this study was more sensitive at detecting any differences in strength present after injury. Participants performed 108 maximum contractions in total, far more than other similar trials [10,14]. Exertional testing could also explain why trends seen on concentric testing may become statistically significant when more demanding 'mixed' concentric and eccentric protocol are used [15]. In addition, a range of isokinetic speeds (60°/sec, 180°/sec and 300°/sec) were used since existing research is inconclusive with respect to the optimal speed for testing, with some suggesting slower speeds [11,13] and others suggesting speeds of 180°/sec or faster as these are thought to be closer to the speed of muscle contraction during sporting activity [34]. In the current study, most of the differences between groups were noticed at the slower speed of 60°/sec, similar to previous research [11]. These contrasting results across isokinetic trials after hamstring injury are not easily explained, but possibly reflect the multifactorial and heterogenous nature of hamstring injuries. It is likely that many other factors need to be considered to get a more complete impression of the 'deficits' that may be present after injury. In addition, methodological differences across trials including the dynamometer brand used, dynamometer speed, mode and testing position, as well as sex and sport of the study population, make comparisons between trials questionable.

### Limitations

It could be argued that the absence of eccentric measurements in this study was a limitation, however concentric testing is safer and was able to identify changes in muscle strength after injury. The amount of muscle soreness associated with eccentric muscle testing may in fact reduce subject compliance and hinder the ability to perform maximal contractions, particularly in subjects who have been previously injured. Reduced compliance with eccentric strengthening programmes has previously been reported in the literature [35]. The retrospective nature of this study does not allow conclusions to be made regarding cause and effect. It is not possible to determine if the weakness observed was present before injury, or developed as a consequence of the injury. Further prospective trials are needed to examine this issue. Due to the lack of access to medical files, the diagnosis of hamstring injury in this study was dependent on the players' own report – which increased the risk of misdiagnosis due to the other possible causes of posterior thigh pain, as well as meaning the incidence of injury may have been significantly under reported [36].  However, retrospective recall has been shown to be accurate in recalling the number of injuries and the body regions injured, albeit not the exact diagnosis [37]. The exact length of time since injury was not examined in this study, which could influence findings and should be recorded in future similar studies. Fatigue must also be taken into account as 108 contractions could have had a substantial effect on the later contractions produced. To minimise the effects of potential fatigue a rest period of 90 seconds was allowed between sets and 180 seconds between each speed. The severity of injury may have been lower in this study than in some previous studies [13], since the average length of time missed was 3.5 weeks. Significant strength differences were still observed in these 'mild' injuries that were 'recovered' however, which we believe is related to the sensitive, demanding protocol used. Finally, many of the differences noted were only statistically significant for some isokinetic parameters, at some of the speeds, which may be related to the small sample size and the small number (n = 15) of injured subjects.

### Implications

The significance of these changes in isokinetic variables, particularly the reduced strength ratios, is that they have been proposed to be risk factors for the development of future hamstring injury in some prospective trials [11,12,34,38,39], albeit not all of them [10,30]. This may be clinically relevant as previous research has demonstrated that persistance of muscle strength abnormalities may give rise to recurrent injuries [16]. Furthermore, 'correction' of isokinetic parameters may reduce injury recurrence [16,18]. This is not to suggest, however, that all players after hamstring injury present with the same 'deficits', and require the same intervention. For example, one previous study [10] found that athletes with a history of hamstring injury had increased, rather than decreased, hamstring strength after injury. In addition, there is evidence of "inter-individual dispersion" of isokinetic results after injury [15,27], so that not all players post-hamstring injury appear to have the exact same impairments of strength. Therefore, treatment programmes should still be matched to the requirements of the individual player. Recovery of hamstring strength may be one aspect of rehabilitation, but it is unlikely to be the only factor which requires consideration [7,8].

## Conclusion

Gaelic footballers with a history of previous hamstring injury had reduced hamstring muscle strength on isokinetic assessment. The injuries were most common on the dominant (kicking) limb. Analysing the muscle strength of athletes after injury requires consideration of a suitable control population, to ensure any 'deficit' present after injury is accurately identified. Otherwise, inherent variability, for example due to dominance, may lead to misleading conclusions. Accurate identification of changes in muscle strength after injury are required for designing appropriate rehabilitation programmes.

## Competing interests

The author(s) declare that they have no competing interests.

## Authors' contributions

KOS was involved in conception and design of the study, data analysis and interpretation, as well as drafting and editing the final document for publication. BOC was involved in conception and design of the study, data collection, data analysis, as well as drafting and editing the final document for publication. KOC was involved in conception and design of the study, data collection, data analysis, as well as drafting and editing the final document for publication. AS was involved in data collection, data interpretation, as well as drafting and editing the final document for publication.

## Pre-publication history

The pre-publication history for this paper can be accessed here:


